# Supplementary material for: Cultural adaptation of the person-centered maternity care scale at governmental health facilities in Cambodia
Source: PLoS One. 2023 Jan 3;18(1):e0265784. doi: 10.1371/journal.pone.0265784 (PMC9810154; doi:10.1371/journal.pone.0265784)
Supplement: S1 Table — (DOCX) [file pone.0265784.s002.docx]

S1 Table. Khmer translation revision process

| # | Original Question | Initial translation Version 1 | Khmer version2  used in 1st round of CIs | English back-translation (ver.2) | Final version | English back-translation  (final version) |  |
| --- | --- | --- | --- | --- | --- | --- | --- |
| 1 | How did you feel about the amount of time you waited? Would you say it was very short, somewhat short, somewhat long, or very long? | តើអ្នកគិតយ៉ាងម៉េចចំពោះពេលវេលានៃការរង់ចាំ? តើអ្នកគិតថាវាខ្លីណាស់ ខ្លីបង្គួរ យូរបង្គួរ ឬយូរណាស់? | តើអ្នកមានអារម្មណ៍យ៉ាងណាចំពោះចំនួននៃពេលវេលាដែលអ្នករង់ចាំក្នុងការទទួលការថែទាំ? | How did you feel about the amount of time you waited to receive care? | តើតាំងពីពេលអ្នកចូលមកមន្ទីពេទ្យរហូតបានទទួលការថែទាំ,អ្នកបានចាំយូរឬឆាប់？ | Did you feel to wait long or short from when you arrived to when you received care? |  |
| 2 | During your time in the health facility did the doctors, nurses, or other health care providers introduce themselves to you when they first came to see you? | ក្នុងអំឡុងពេលអ្នកនៅមន្ទីរពេទ្យ តើគ្រូពេទ្យនិងបុគ្គលិកផ្សេងទៀតបានណែនាំខ្លួនគេដល់អ្នកនៅពេលដែលគេជួបអ្នកលើកដំបូងឬទេ? | គ្រូពេទ្យនិងបុគ្គលិកផ្សេងទៀតបានស្វាគមន៍អ្នកទេ នៅពេលដែលគេជួបអ្នកលើកដំបូងឬទេ? | During your time in the health facility did the doctors, nurses, or other health care providers welcome you when they first came to see you? | តើអំឡុងពេលអ្នកនៅក្នុងមន្ទីរពេទ្យ/មណ្ឌលសុខភាព，ក្រុមគ្រូពេទ្យបានណែនាំខ្លួន　ពេលពួកគាត់បានជួបអ្នកលើកដំបូងដែរឬទេ?ឧទាហរណ៍ ប្រាប់ឈ្មោះនិងជំនាញរបស់ពួកគេទេ? | During your time in the health facility did the medical staffs introduce themselves to you when they first came to see you? For example, their name or profession. |  |
|  |  |  |  |  | តើក្រុមគ្រូពេទ្យ បានប្រាប់អ្នកថាគាត់គឺជាអ្នកជួយសម្រាលកូនអោយអ្នកដែរឬទេ? | Did the medical staffs tell you they are the one who support your childbirth? |  |
| 3 | Did the doctors, nurses, or other health care providers call you by your name? | តើគ្រូពេទ្យ ឬបុគ្គលិក ដទៃទៀតនៅ បានហៅអ្នកតាមឈ្មោះទេ? | តើគ្រូពេទ្យ ឬបុគ្គលិក ដទៃទៀត បានហៅអ្នកដោយសមរម្យដោយមិនរើសអើងឬមើលងាយអ្នកទេ?​ | Did the doctors, nurses, or other health care providers call you appropriately by not discriminating or looking down on you? | តើក្រុមគ្រូពេទ្យបានហៅអ្នកតាមឈ្មោះដែរឬទេ? | Did the medical staffs call you by your name? |  |
|  |  |  |  |  | តើគ្រូពេទ្យឬបុគ្គលិក ដទៃទៀត បានហៅអ្នកដោយសមរម្យឬទេ? | Did the medical staffs call you appropriately? |  |
| 6 | During examinations in the labor room, were you covered up with a cloth or blanket or screened with a curtain so that you did not feel exposed? | ក្នុងអំឡុងពេលពិនិត្យក្នុងបន្ទប់ឈឺពោះ សម្រាល តើអ្នកត្រូវបានគេគ្របដោយក្រណាត់ ឬភួយ ឬ បាំងដោយវាំងននដើម្បីកុំអោយ អ្នកដទៃមើលឃើញឬទេ? | ក្នុងអំឡុងពេលពិនិត្យក្នុងបន្ទប់ឈឺពោះ សម្រាល តើអ្នកត្រូវបានគេ បាំងដើម្បីកុំអោយ អ្នកដទៃមើលឃើញឬទេ? | During examinations in the labor room, were you covered up that no one else could see? | អំឡុងពេលពិនិត្យនៅក្នុងបន្ទប់ឈឺពោះសម្រាលកូន (ឧទាហរណ៍ការពិនិត្យស្បូន) តើអ្នកគិតថាអ្នកត្រូវបានគេបាំង ដោយគេព្យួរក្រណាត់រឺភួយរឺបិទជាមួយវាំងននទេ? | During examinations in the labor room (for example, pelvic examination), were you covered up with a cloth or blanket or screened with a curtain? |  |
| 7 | Do you feel like your health information was or will be kept confidential at this facility? | តើអ្នកគិតថាថាពត៌មានសុខភាព របស់អ្នក ត្រូវបានគេរក្សាជាការសំងាត់ទេ? | តើអ្នកគិតថាពត៌មានសុខភាព របស់អ្នក ត្រូវបានគេរក្សាជាការសំងាត់ទេ? | Do you feel like your health information was kept confidential? | តើអ្នកគិតថា ពត៌មានសុខភាពរបស់អ្នក ត្រូវបានក្រុមគ្រូពេទ្យរក្សាជាការសម្ងាត់​ ដែរឬទេ? | Do you feel like your health information was kept confidential at this facility? For example, the information on the medical record. |  |
|  |  |  |  |  |  |  |  |
|  |  |  |  |  |  |  |  |
| 8 | Did you feel like the doctors, nurses or other staff at the facility involved you in decisions about your care? | តើអ្នកគិតថាគ្រូពេទ្យ ឬបុគ្គលិកដទៃទៀតបានអោយអ្នកចូលរួមក្នុងការសម្រេចចិត្តទាក់ទងនឹង ការថែទាំរបស់អ្នកឬទេ? | តើអ្នកគិតថាគ្រូពេទ្យ ឬបុគ្គលិកដទៃទៀតបានអោយអ្នកចូលរួមក្នុងការសម្រេចចិត្តទាក់ទងនឹង ការថែទាំរបស់អ្នកឬទេ? | Did you feel like the doctors, nurses or other staff at the facility involved you in decisions about your care? | ក្នុងការសម្រាលកូន លើកនេះ, តើ ក្រុមគ្រូពេទ្យបានសួរយោបល់ឬការសម្រេចចិត្តរបស់អ្នកដែរឬទេ？ឧទាហរណ៍តើអ្នកអាចសម្រេចចិត្តដោយខ្លួនឯងថាចង់សម្រាលកូនដោយធម្មជាតិឬវះកាត់បានទេ | Did you feel like the medical staffs at the facility considered your ideas in decisions about your care? For example, can you decide for yourself whether you want to have a natural or caesarean section? |  |
| 9 | Did the doctors, nurses or other staff at the facility ask your permission/consent before doing procedures on you? | តើគ្រូពេទ្យ ឬ បុគ្គលិកដទៃទៀត បានសុំការអនុញ្ញាតិអ្នក/ការយល់ព្រមរបស់អ្នកមុន ពេលពិនិត្យឬទេ? | តើគ្រូពេទ្យ ឬ បុគ្គលិកដទៃទៀត បានសុំការអនុញ្ញាតិអ្នក/ការយល់ព្រមរបស់អ្នកមុន ពេលពិនិត្យឬទេ? | Did the doctors, nurses or other staff at the facility ask your permission/consent before doing procedures on you? | តើមុនពេលពិនិត្យដូចជាពិនិត្យស្បូនជាដើម ក្រុមគ្រូពេទ្យបានសុំការអនុញ្ញាត / ការយល់ព្រម ពីអ្នកដែរឬទេ? | Did the medical staffs at the facility ask your permission/consent before doing procedures on you? For example, pelvic examination and episiotomy? |  |
| 10 | During the delivery, do you feel like you were able to be in the position of your choice? | ក្នុងអំឡុងពេលសម្រាល តើអ្នកគិតថា អ្នកអាចស្ថិតនៅក្នុងឥរិយាបថដែលជាជម្រើសរបស់អ្នកឬទេ? | តើពេលសម្រាលអ្នកគិតថា​ អ្នកស្ថិតក្នុង​ឥរិយាបថដែលងាយស្រួលសម្រាលកូនឬទេ ? | During the delivery, do you think you were in a comfortable delivery position? | ក្នុងអំឡុងពេលឈឺពោះសម្រាលកូន, តើអ្នកគិតថា អ្នកអាចធ្វើចលនាបានដោយសេរីដេរឬទេ | During the delivery, do you feel like you were able to be in your favorite free position? |  |
|  |  |  |  |  | តើពេលសម្រាល អ្នកបានគេងបញ្ឈរជង្គង់មែនទេ | Did you deliver in the supine position? |  |
| 12 | Did the doctors and nurses explain to you why they were doing examinations or procedures on you? | តើគ្រូពេទ្យពន្យល់អ្នកថាតើហេតុអ្វីបាន ជាពួកគេកំពុងពិនិត្យអ្នកឬធ្វើសកម្មភាពណាមួយលើអ្នកឬទេ? | តើគ្រូពេទ្យពន្យល់អ្នកថាតើហេតុអ្វីបាន ជាពួកគេកំពុងពិនិត្យអ្នកឬធ្វើសកម្មភាពណាមួយលើអ្នកឬទេ? | Did the doctors and nurses explain to you why they were doing examinations or procedures on you? | តើក្រុមគ្រូពេទ្យបានពន្យល់អ្នកពីគោលបំណងនិងមូលហេតុដែលគេធ្វើតេស្តឬពិនិត្យអ្នកទេ?　ឧទាហរណ៍ពេលពិនិត្យស្បូននិងស្តាប់បេះដូងកូន | Did the medical staffs explain to you the objectives or reasons why they were doing examinations or procedures on you? For example, pelvic examination or fetal heart rate monitoring |  |
| 14 | Did the doctors and nurses at the facility talk to you about how you were feeling? | តើគ្រូពេទ្យបានជជែកជាមួយអ្នកថា តើអ្នកមានអារម្មណ៍ដូចម្តេចទេ? | តើគ្រូពេទ្យបានសួរអ្នកពីអារម្មណ៍អ្នកទេ? | Did the doctors and nurses at the facility talk to you about how you were feeling? | តើក្រុមគ្រូពេទ្យ បានសួរអ្នកថា តើអ្នកស្រួលខ្លួន ហើយឬនៅ | Did the medical staffs at the facility talk to you about how you were feeling (Physical)? |  |
|  |  |  |  |  | តើក្រុមគ្រូពេទ្យ បានសួរអ្នក ពីអារម្មណ៍របស់អ្នក ដែរឬទេ? | Did the medical staffs at the facility talk to you about how you were feeling (Psychological)? |  |
| 19 | When you needed help, did you feel the doctors, nurses or other staff at the facility paid attention? | នៅពេលដែលអ្នកត្រូវការជំនួយ តើអ្នកគិតថា គ្រូពេទ្យ ឬបុគ្គលិក ដទៃទៀត យកចិត្តទុកដាក់ ចំពោះអ្នក ឬទេ? | នៅពេលដែលអ្នកមានបញ្ហា តើអ្នកគិតថា គ្រូពេទ្យ ឬបុគ្គលិកដទៃទៀត យកចិត្តទុកដាក់ ចំពោះអ្នក ឬទេ? | When you have a problem, did you feel doctors or other staff care about you? | នៅពេលអ្នកត្រូវការជំនួយ　តើអ្នកគិតថា　ក្រុមគ្រូពេទ្យបានយល់ពីតម្រូវការរបស់អ្នកទេ? | When you needed help, did you feel the medical staffs at the facility respond to what you need? |  |
