# Supplementary material for: Cultural adaptation of the person-centered maternity care scale at governmental health facilities in Cambodia
Source: PLoS One. 2023 Jan 3;18(1):e0265784. doi: 10.1371/journal.pone.0265784 (PMC9810154; doi:10.1371/journal.pone.0265784)
Supplement: S2 Table — (DOCX) [file pone.0265784.s003.docx]

S2 Table. Kh-PCMC scale

| Back translation of Kh-PCMC scale | | Khmer translation of the PCMC scale |
| --- | --- | --- |
| SCALE TO MEASURE PERSON-CENTERED MATERNITY CARE DURING LABOR AND DELIVERY | | រង្វាស់សម្រាប់វាស់ការថែទាំម្តាយមជ្ឈមណ្ឌល ក្នុងអំឡុងពេលឈឺពោះសម្រាល និងពេលសម្រាលនៅប្រទេសកម្ពុជា |
| Now I am going to ask you some questions about your experiences in the health facility during your last delivery. Remember that all the questions in this section refer specifically to the time you were in the health facility for this last delivery. Also, know that everything you tell me is confidential and will not be shared with the health facility.” | | “ឥឡូវនេះ ខ្ញុំសូមសួរអ្នកអំពីបទពិសោធន៍នៅមូលដ្ឋានសុខាភិបាលក្នុងអំឡុង ពេលសម្រាលកូនចុងក្រោយរបស់អ្នក។ សូមចងចាំថា សំនួរទាំងអស់នៅក្នុងផ្នែក នេះសំដៅផ្ទាល់ទៅលើពេលវេលាដែលអ្នកនៅមូលដ្ឋានសុខាភិបាលសម្រាប់ការ សម្រាលកូនចុងក្រោយ។ ជាមួយគ្នានេះដែរ រាល់ពត៌មានទាំងអស់របស់អ្នក គឺ ត្រូវបានរក្សាជាការសម្ងាត់ ហើយនឹងមិនចែកចាយជាមួយមូលដ្ឋានសុខាភិបាលនោះទេ។ |
| #1. Did you feel to wait long or short from when you arrived to when you received care? | | តើតាំងពីពេលអ្នកចូលមកមន្ទីពេទ្យរហូតបានទទួលការថែទាំ,អ្នកបានចាំយូរឬឆាប់？ |
|  | 0 very short | ខ្លីណាស់ |
|  | 1 Somewhat short | ខ្លីបង្គួរ |
|  | 2 Somewhat long | យូរបង្គួរ |
|  | 3 very long | យូរណាស់ |
|  |  |  |
| #2. During your time in the health facility did the medical staff introduce themselves to you when they first came to see you? For example, their name or profession. | | តើអំឡុងពេលអ្នកនៅក្នុងមន្ទីរពេទ្យ/មណ្ឌលសុខភាព，ក្រុមគ្រូពេទ្យបានណែនាំខ្លួន　ពេលពួកគាត់បានជួបអ្នកលើកដំបូងដែរឬទេ?ឧទាហរណ៍ ប្រាប់ឈ្មោះនិងជំនាញរបស់ពួកគេទេ? |
|  | 0 No, none of them | ទេមិនមានទេ |
|  | 1 Yes, a few of them | ចាសមានម្នាក់ឬពីរនាក់ |
|  | 2 Yes, most of them | ចាសភាគច្រើន |
|  | 3 Yes, all of them | ចាសទាំងអស់គ្នា |
|  |  |  |
| Back translation of Kh-PCMC scale | | Khmer translation of the PCMC scale |
| Now I will ask you some questions about how you were treated at the health facility. Tell me if the following things happened all the time, most of the time, a few times, or it never happened. You can say a few times if it happened one or two times, and most of the time will be if it happened 3 or more times, but not always. For some questions I will ask specifically if something occurred during labor, delivery, or after delivery. If I do not specify please answer based on your experiences during the entire time you were in the facility from labor till discharge. | | “ឥឡូវនេះខ្ញុំសូមសួរអ្នកថាតើអ្នកត្រូវបានគេថែទាំអ្នកដោយរបៀបណានៅមូលដ្ឋាន សុខាភិបាល។ សូមប្រាប់ខ្ញុំប្រសិនបើរឿងទាំងអស់កើតមានគ្រប់ពេល ភាគច្រើន ពីរបីដង ឬមិនដែលកើតមានឡើង។ អ្នកអាចនិយាយថាម្តងឬពីរដង ប្រសិនបើវាកើតឡើងម្តងឬពីរដង និង ភាគច្រើន ប្រសិនបើវាកើតឡើងបីដងឬច្រើនជាងនេះ តែមិនមែនជានិច្ចកាល។ សម្រាប់ សំនួរខ្លះ ខ្ញុំនឹងសួរបញ្ជាក់ប្រសិន បើកើតមានក្នុងអំឡុងពេលឈឺពោះសម្រាល សម្រាល ឬបន្ទាប់ពីសម្រាល។ ប្រសិនបើខ្ញុំមិន សួរបញ្ជាក់ សូមឆ្លើយតាមបទពិសោធន៍របស់ អ្នកក្នុង អំឡុងពេលអ្នកនៅមូលដ្ឋានសុខាភិបាល រហូតដល់ចេញពីមូលដ្ឋានសុខាភិបាល។ |
| #3.1. Did the medical staff call you by your name? | | តើក្រុមគ្រូពេទ្យបានហៅអ្នកតាមឈ្មោះដែរឬទេ? |
|  | 0 No, never | ទេ មិនដែលទេ |
|  | 1 Yes, a few times | ចាស ម្តងឬពីរដង |
|  | 2 Yes, most of the time | ចាស ភាគច្រើន |
|  | 3 Yes, all the time | ចាស គ្រប់ពេល |
|  |  |  |
| #3.2. Did the medical staff call you appropriately? | | តើគ្រូពេទ្យឬបុគ្គលិក ដទៃទៀត បានហៅអ្នកដោយសមរម្យឬទេ? |
|  | 0 No, never | ទេ មិនដែលទេ |
|  | 1 Yes, a few times | ចាស ម្តងឬពីរដង |
|  | 2 Yes, most of the time | ចាស ភាគច្រើន |
|  | 3 Yes, all the time | ចាស គ្រប់ពេល |
|  |  |  |
| #4. Did the medical staff at the facility treat you with respect? | | តើក្រុមគ្រូពេទ្យបានថែទាំអ្នកដោយយកចិត្តទុកដាក់និងការគោរពឬទេ? |
|  | 0 No, never | ទេ មិនដែលទេ |
|  | 1 Yes, a few times | ចាស ម្តងឬពីរដង |
|  | 2 Yes, most of the time | ចាស ភាគច្រើន |
|  | 3 Yes, all the time | ចាស គ្រប់ពេល |

| Back translation of Kh-PCMC scale | | Khmer translation of the PCMC scale |
| --- | --- | --- |
| #5. Did the medical staff at the facility treat you in a friendly manner? | | តើក្រុមគ្រូពេទ្យ បានថែទាំអ្នកដោយរាក់ទាក់ស្និតស្នាលដែរឬទេ? |
|  | 0 No, never | ទេ មិនដែលទេ |
|  | 1 Yes, a few times | ចាស ម្តងឬពីរដង |
|  | 2 Yes, most of the time | ចាស ភាគច្រើន |
|  | 3 Yes, all the time | ចាស គ្រប់ពេល |
|  |  |  |
| #6. During examinations in the labor room (for example, pelvic examination), were you covered up with a cloth or blanket or screened with a curtain? | | អំឡុងពេលពិនិត្យនៅក្នុងបន្ទប់ឈឺពោះសម្រាលកូន (ឧទាហរណ៍ការពិនិត្យស្បូន) តើអ្នកគិតថាអ្នកត្រូវបានគេបាំង ដោយគេព្យួរក្រណាត់រឺភួយរឺបិទជាមួយវាំងននទេ? |
|  | 0 No, never | ទេ មិនដែលទេ |
|  | 1 Yes, a few times | ចាស ម្តងឬពីរដង |
|  | 2 Yes, most of the time | ចាស ភាគច្រើន |
|  | 3 Yes, all the time | ចាស គ្រប់ពេល |
|  |  |  |
| #7. Do you feel like your health information was kept confidential at this facility? For example, the information on the medical record. | | តើអ្នកគិតថា ពត៌មានសុខភាពរបស់អ្នក ត្រូវបានក្រុមគ្រូពេទ្យរក្សាជាការសម្ងាត់​ ដែរឬទេ? ឧទាហរណ៍ព័ត៌មានរបស់អ្នកក្នុងរបាយការណ៍ពេទ្យ |
|  | 0 No, never | ទេ មិនដែលទេ |
|  | 1 Yes, a few times | ចាស ម្តងឬពីរដង |
|  | 2 Yes, most of the time | ចាស ភាគច្រើន |
|  | 3 Yes, all the time | ចាស គ្រប់ពេល |
|  | 4 Did not know it was kept confidential | មិនដឹងថាគេលាក់ការសម្ងាត់ឬអត់ទេ |

| Back translation of Kh-PCMC scale | | Khmer translation of the PCMC scale |
| --- | --- | --- |
| #8. Did you feel like the medical staff at the facility considered your ideas in decisions about your care? For example, can you decide for yourself whether you want to have a natural or caesarean section? | | ក្នុងការសម្រាលកូន លើកនេះ, តើ ក្រុមគ្រូពេទ្យបានសួរយោបល់ឬការសម្រេចចិត្តរបស់អ្នកដែរឬទេ？ឧទាហរណ៍តើអ្នកអាចសម្រេចចិត្តដោយខ្លួនឯងថាចង់សម្រាលកូនដោយធម្មជាតិឬវះកាត់បានទេ? |
|  | 0 No, never | ទេ មិនដែលទេ |
|  | 1 Yes, a few times | ចាស ម្តងឬពីរដង |
|  | 2 Yes, most of the time | ចាស ភាគច្រើន |
|  | 3 Yes, all the time | ចាស គ្រប់ពេល |
|  | 4 preferable to follow doctors | សម្រេចតាមពេទ្យល្អជាង |
|  |  |  |
| #9. Did the medical staff at the facility ask your permission/consent before doing procedures on you? For example, pelvic examination and episiotomy? | | តើមុនពេលពិនិត្យដូចជាពិនិត្យស្បូនជាដើម ក្រុមគ្រូពេទ្យបានសុំការអនុញ្ញាត / ការយល់ព្រម ពីអ្នកដែរឬទេ? |
|  | 0 No, never | ទេ មិនដែលទេ |
|  | 1 Yes, a few times | ចាស ម្តងឬពីរដង |
|  | 2 Yes, most of the time | ចាស ភាគច្រើន |
|  | 3 Yes, all the time | ចាស គ្រប់ពេល |
|  |  |  |
| #10. During the delivery, do you feel like you were able to be in your favorite free position? | | ក្នុងអំឡុងពេលឈឺពោះសម្រាលកូន, តើអ្នកគិតថា អ្នកអាចធ្វើចលនាបានដោយសេរីដេរឬទេ ? |
|  | 0 No, never | ទេ មិនដែលទេ |
|  | 1 Yes, for a short time | ចាស ម្តងឬពីរដង |
|  | 2 Yes, most of the time | ចាស ភាគច្រើន |
|  | 3 Yes, all the time | ចាស គ្រប់ពេល |
|  | 4 No choice other than following doctors​ | គ្មានជម្រើស ក្រៅពីធ្វើតាម គ្រួពេទ្យ |
|  |  |  |
| #11. Did the medical staffs at the facility speak to you in a language you could understand? | | តើក្រុមគ្រូពេទ្យបាននិយាយជាមួយអ្នកដោយប្រើពាក្យសាមញ្ញៗដែលអ្នកអាចយល់ន័យបានឬទេ? |
|  | 0 No, never | ទេ មិនដែលទេ |
|  | 1 Yes, a few times | ចាស ម្តងឬពីរដង |
|  | 2 Yes, most of the time | ចាស ភាគច្រើន |
|  | 3 Yes, all the time | ចាស គ្រប់ពេល |

| Back translation of Kh-PCMC scale | | Khmer translation of the PCMC scale |
| --- | --- | --- |
| #12. Did the medical staff explain to you the objectives or reasons why they were doing examinations or procedures on you? For example, pelvic examination or fetal heart rate monitoring. | | តើក្រុមគ្រូពេទ្យបានពន្យល់អ្នកពីគោលបំណងនិងមូលហេតុដែលគេធ្វើតេស្តឬពិនិត្យអ្នកទេ?　ឧទាហរណ៍ពេលពិនិត្យស្បូននិងស្តាប់បេះដូងកូន |
|  | 0 No, never | ទេ មិនដែលទេ |
|  | 1 Yes, a few times | ចាស ម្តងឬពីរដង |
|  | 2 Yes, most of the time | ចាស ភាគច្រើន |
|  | 3 Yes, all the time | ចាស គ្រប់ពេល |
|  |  |  |
| #13. Did the medical staff explain to you why they were giving you any medicine? | | តើក្រុមគ្រូពេទ្យ បានពន្យល់ពីមូលហេតុថា ហេតុអ្វីបានជាពួកគេផ្តល់ថ្នាំណាមួយអោយអ្នកឬទេ? |
|  | 0 No, never | ទេ មិនដែលទេ |
|  | 1 Yes, a few times | ចាស ម្តងឬពីរដង |
|  | 2 Yes, most of the time | ចាស ភាគច្រើន |
|  | 3 Yes, all the time | ចាស គ្រប់ពេល |
|  | 4 Did not get any medicine | មិនដែលបានទទួលថ្នាំណាមួយទេ |
|  |  |  |
| #14.1. Did the medical staff at the facility talk to you about how you were feeling? (Physical) | | តើក្រុមគ្រូពេទ្យ បានសួរអ្នកថា តើអ្នកស្រួលខ្លួន ហើយឬនៅ? |
|  | 0 No, never | ទេ មិនដែលទេ |
|  | 1 Yes, a few times | ចាស ម្តងឬពីរដង |
|  | 2 Yes, most of the time | ចាស ភាគច្រើន |
|  | 3 Yes, all the time | ចាស គ្រប់ពេល |
|  |  |  |
| #14.2. Did the medical staff at the facility talk to you about how you were feeling? (Psychological) | | តើក្រុមគ្រូពេទ្យ បានសួរអ្នក ពីអារម្មណ៍របស់អ្នក ដែរឬទេ? |
|  | 0 No, never | ទេ មិនដែលទេ |
|  | 1 Yes, a few times | ចាស ម្តងឬពីរដង |
|  | 2 Yes, most of the time | ចាស ភាគច្រើន |
|  | 3 Yes, all the time | ចាស គ្រប់ពេល |

| Back translation of Kh-PCMC scale | | Khmer translation of the PCMC scale |
| --- | --- | --- |
| #15. Did the medical staff at the facility try to understand your anxieties and fears? | | តើក្រុមគ្រូពេទ្យបានព្យាយាមយល់ពីការព្រួយបារម្ភនិងការភ័យខ្លាចរបស់អ្នកទេ? |
|  | 0 No, never | ទេ មិនដែលទេ |
|  | 1 Yes, a few times | ចាស ម្តងឬពីរដង |
|  | 2 Yes, most of the time | ចាស ភាគច្រើន |
|  | 3 Yes, all the time | ចាស គ្រប់ពេល |
|  | 4 I did not have any anxieties or fears | មិនមានការព្រួយបារម្ភ ឬភ័យខ្លាចទេ |
|  |  |  |
| #16. Did you feel you could ask t the medical staffs at the facility any questions you had? | | តើអ្នកគិតថា អ្នកអាចសួរសំនួរណាមួយទៅក្រុមគ្រូពេទ្យ ដោយស្រួលដែរឬទេ？ |
|  | 0 No, never | ទេ មិនដែលទេ |
|  | 1 Yes, a few times | ចាស ម្តងឬពីរដង |
|  | 2 Yes, most of the time | ចាស ភាគច្រើន |
|  | 3 Yes, all the time | ចាស គ្រប់ពេល |
|  |  |  |
| #17. Were you allowed to have someone you wanted to stay with you during labor? | | តើអ្នកត្រូវបានអនុញ្ញាតឱ្យនៅជាមួយនរណាម្នាក់ដែលអ្នកចង់នៅជាមួយក្នុងបន្ទប់ឈឺពោះសម្រាលដែរឬទេ? |
|  | 0 No, never | ទេ មិនដែលទេ |
|  | 1 Yes, a few times | ចាស ម្តងឬពីរដង |
|  | 2 Yes, most of the time | ចាស ភាគច្រើន |
|  | 3 Yes, all the time | ចាស គ្រប់ពេល |
|  | 4 I did not want someone to stay with me | ញុំមិនត្រូវការនរណាម្នាក់មកនៅជាមួយខ្ញុំទេ |
|  |  |  |
| #18. Were you allowed to have someone you wanted to stay with you during delivery? | | តើអ្នកត្រូវបានអនុញ្ញាតឱ្យនៅជាមួយនរណាម្នាក់ដែលអ្នកចង់នៅជាមួយក្នុងបន្ទប់សម្រាលដែឬទេ? |
|  | 0 No, never | ទេ មិនដែលទេ |
|  | 1 Yes, a few times | ចាស ម្តងឬពីរដង |
|  | 2 Yes, most of the time | ចាស ភាគច្រើន |
|  | 3 Yes, all the time | ចាស គ្រប់ពេល |
|  | 4 I did not want someone to stay with me | ខ្ញុំមិនត្រូវការនរណាម្នាក់មកនៅជាមួយខ្ញុំទេ |

| Back translation of Kh-PCMC scale | | | Khmer translation of the PCMC scale |
| --- | --- | --- | --- |
| #19. When you needed help, did you feel the medical staff at the facility respond to needs? | | | នៅពេលអ្នកត្រូវការជំនួយ　តើអ្នកគិតថា　ក្រុមគ្រូពេទ្យបានយល់ពីតម្រូវការរបស់អ្នកទេ? |
|  | 0 No, never | | ទេ មិនដែលទេ |
|  | 1 Yes, a few times | | ចាស ម្តងឬពីរដង |
|  | 2 Yes, most of the time | | ចាស ភាគច្រើន |
|  | 3 Yes, all the time | | ចាស គ្រប់ពេល |
|  |  | |  |
| #20. Do you feel the medical staff did everything they could to help control your pain? | | | នៅពេលអ្នកឈឺពោះ　តើអ្នកគិតថាក្រុមគ្រូពេទ្យបានព្យាយាមជួយកាត់បន្ថយអាការៈនោះដែរឬទេ? |
|  | 0 No, never | | ទេ មិនដែលទេ |
|  | 1 Yes, a few times | | ចាស ម្តងឬពីរដង |
|  | 2 Yes, most of the time | | ចាស ភាគច្រើន |
|  | 3 Yes, all the time | | ចាស គ្រប់ពេល |
|  | 4 No pain | | អត់មានឈឺពោះទេ |
|  |  | |  |
| #21. Did you feel the medical staff shouted at you, scolded, insulted, threatened, or talked to you rudely? | | | តើអ្នកគិតថាក្រុមគ្រូពេទ្យបានស្រែកគំហកដាក់អ្នក ស្តីបន្ទោសអ្នក ជេរប្រមាថអ្នក គម្រាមអ្នក ឬនិយាយ ឈ្លើយ មកកាន់អ្នកដែរឬ្ទទេ? |
|  | 0 No, never | | ទេ មិនដែលទេ |
|  | 1 Yes, once | | ចាស ម្តងឬពីរដង |
|  | 2 Yes, a few times | | ចាស ភាគច្រើន |
|  | 3 Yes, many time | | ចាស គ្រប់ពេល |
|  |  | |  |
| #22. Did you feel like you were treated roughly like pushed, beaten, slapped, pinched, physically restrained, or gagged? | | | តើអ្នកគិតថាត្រូវបានគេរុញ,វាយដំ,វាយទះកំភ្លៀង, ក្តិច,ឃាត់ឃាំងរាងកាយ, ហាមឃាត់មិនឱ្យនិយាយដោយសេរីឬត្រូវគេធ្វើបាបឬទេ? |
|  | 0 No, never | | ទេ មិនដែលទេ |
|  | 1 Yes, once | | ចាស ម្តងឬពីរដង |
|  | 2 Yes, a few times | | ចាស ភាគច្រើន |
|  | 3 Yes, many time | | ចាស គ្រប់ពេល |
|  | |  |  |

| Back translation of Kh-PCMC scale | | Khmer translation of the PCMC scale |
| --- | --- | --- |
| #23. Did the medical staff at the facility ask you or your family for money other than the official cost? | | តើក្រុមគ្រូពេទ្យ សុំលុយទឹកតែ ពីអ្នកឬ គ្រួសារអ្នកឬទេ? |
|  | 0 No, never | ទេ មិនដែលទេ |
|  | 1 Yes, a few times | ចាស ម្តងឬពីរដង |
|  | 2 Yes, most of the time | ចាស ភាគច្រើន |
|  | 3 Yes, all the time | ចាស គ្រប់ពេល |
|  |  |  |
| #24. Do you think there was enough health staff in the facility to care for you? | | តើអ្នកគិតថាមានបុគ្គលិកថែទាំសុខភាពគ្រប់គ្រាន់ ដើម្បីថែទាំអ្នកដែរឬទេ? |
|  | 0 No, never | ទេ មិនដែលទេ |
|  | 1 Yes, a few times | ចាស ម្តងឬពីរដង |
|  | 2 Yes, most of the time | ចាស ភាគច្រើន |
|  | 3 Yes, all the time | ចាស គ្រប់ពេល |
|  |  |  |
| #25. Did you feel the medical staff at the facility took the best care of you? | | តើអ្នកគិតថាក្រុមគ្រូពេទ្យ ថែទាំអ្នកបានយ៉ាងល្អដែរឬទេ? |
|  | 0 No, never | ទេ មិនដែលទេ |
|  | 1 Yes, a few times | ចាស ម្តងឬពីរដង |
|  | 2 Yes, most of the time | ចាស ភាគច្រើន |
|  | 3 Yes, all the time | ចាស គ្រប់ពេល |
|  |  |  |
| #26. Did you feel you could completely trust the medical staff at the facility with regards to your care? | | តើអ្នកគិតថាអ្នកអាច ជឿទុកចិត្ត ក្រុមគ្រូពេទ្យទាំងស្រុងទាក់ទងនឹងការ ថែទាំអ្នកដែរឬទេ? |
|  | 0 No, never | ទេ មិនដែលទេ |
|  | 1 Yes, a few times | ចាស ម្តងឬពីរដង |
|  | 2 Yes, most of the time | ចាស ភាគច្រើន |
|  | 3 Yes, all the time | ចាស គ្រប់ពេល |
|  |  |  |
| #27. Thinking about the labor and postnatal wards, did you feel the health facility was crowded? | | តើអ្នកគិតថាមន្ទីរពេទ្យ / មណ្ឌលសុខភាពមានមនុស្សច្រើនកកកុញនៅបន្ទប់សំរាលកូននិងកន្លែងថែទាំកូនទេ? |
|  | 0 No, never | ទេ មិនដែលទេ |
|  | 1 Yes, once | ចាស ម្តងឬពីរដង |
|  | 2 Yes, a few times | ចាស ភាគច្រើន |
|  | 3 Yes, many time | ចាស ច្រើនដង |

| Back translation of Kh-PCMC scale | | Khmer translation of the PCMC scale |
| --- | --- | --- |
| #28. Thinking about the wards, washrooms and the general environment of the health facility, will you say the facility was very clean, clean, dirty, or very dirty? | | តើ បន្ទប់លាងដៃនិងកន្លែងថែទាំសុខភាពទាំងមូល ស្អាតខ្លាំង,ស្អាត,កខ្វក់ឬកខ្វក់ខ្លាំងមែនទេ? |
|  | 0 Very dirty | កខ្វក់ណាស់ |
|  | 1 Dirty | កខ្វក់ |
|  | 2 Clean | ស្អាត |
|  | 3 Very clean | ស្អាតណាស់ |
|  |  |  |
| #29. Was there water in the facility? | | តើនៅមន្ទីរពេទ្យមានទឹកដែលអាចប្រើបានឬទេ? |
|  | 0 No, never | ទេ មិនដែលទេ |
|  | 1 Yes, a few times | ចាស ម្តងឬពីរដង |
|  | 2 Yes, most of the time | ចាស ភាគច្រើន |
|  | 3 Yes, all the time | ចាស គ្រប់ពេល |
|  |  |  |
| #30. Was there electricity in the facility? | | តើនៅមន្ទីរពេទ្យ មានភ្លើងអគ្គិសនីដែលអាចប្រើបាន ឬទេ? |
|  | 0 No, never | ទេ មិនដែលទេ |
|  | 1 Yes, a few times | ចាស ម្តងឬពីរដង |
|  | 2 Yes, most of the time | ចាស ភាគច្រើន |
|  | 3 Yes, all the time | ចាស គ្រប់ពេល |
|  |  |  |
| #31. In general, did you feel safe in the health facility? | | ជាទូទៅ តើគិតថានៅមន្ទីរពេទ្យមាន សុវត្ថិភាព ដែរឬទេ? |
|  | 0 No, never | ទេ មិនដែលទេ |
|  | 1 Yes, a few times | ចាស ម្តងឬពីរដង |
|  | 2 Yes, most of the time | ចាស ភាគច្រើន |
|  | 3 Yes, all the time | ចាស គ្រប់ពេល |
|  |  |  |
| *Excluded from final India scale: #15 (Support anxiety), #27 (Crowding), #29 (Water), #30 (Electricity)  Excluded from final Kenya scale: #23 (Bribes) | | |
