# Supplementary material for: Cultural adaptation of the person-centered maternity care scale at governmental health facilities in Cambodia
Source: PLoS One. 2023 Jan 3;18(1):e0265784. doi: 10.1371/journal.pone.0265784 (PMC9810154; doi:10.1371/journal.pone.0265784)
Supplement: Attachment — Submitted filename: Comments.doc [file pone.0265784.s005.doc]

This study has thrown light up on some of the ignored areas of healthcare delivery system. This has got immense role in improving access and utilization of the public healthcare system by the citizens. This qualitative research is expected to help the national policy makers to rectify the loopholes if any.
