# Supplementary material for: Cultural adaptation of the person-centered maternity care scale at governmental health facilities in Cambodia
Source: PLoS One. 2023 Jan 3;18(1):e0265784. doi: 10.1371/journal.pone.0265784 (PMC9810154; doi:10.1371/journal.pone.0265784)
Supplement: Attachment — Submitted filename: Response to Reviewers.docx [file pone.0265784.s006.docx]

RESPONSES TO THE REVIEWERS’ COMMENTS

We are extremely grateful to the editor and reviewers for their comments on the initial version of the manuscript. We have revised the manuscript based on those useful comments. Please refer to the following point-by-point-based responses to the respective comments, along with revised manuscript. Please note that the changes in the revised manuscript with track change have been highlighted in red.

[Response to Reviewer #1]

| Line  page | Comment | Response |
| --- | --- | --- |
| line195  page12 | How did you justify the sample size? What respondent to item ration was followed? | Thanks for this comment. Please see “While an earlier study recommended not more than 10 for each round [25] as the total number of respondents for cognitive interviewing, others recommended 10-30 [40] or at least 15 [43]. Therefore, this study recruited 20 postpartum women for cognitive interviewing.” (Line184-187)  For your information, there are several published papers from this journal using cognitive interview.  http://journals.plos.org/plosone/article?id=10.1371/journal.pone.0186745  http://journals.plos.org/plosone/article?id=10.1371/journal.pone.0069564  http://journals.plos.org/plosone/article?id=10.1371/journal.pone.0076305  http://journals.plos.org/plosone/article?id=10.1371/journal.pone.0178043 |
| line219  page14 | For finalization of this translated tool we need to look at reliability, validity and factor analysis. | Thanks for important clarification. This study serves as the qualitative analysis part of the entire of process of developing a scale in which a previously validated tool is adapted to Cambodian context. Based on this qualitative study, quantitative study will be conducted for the purpose of assessing reliability and validity of the PCMC scale and further published separately from this study. Note that refining the questionnaire using cognitive interviewing is the key foundation for assessing reliability and validity of PCMC scale. |
| line224  page14 | Were there any mothers who had adverse perinatal outcomes? i.e stillbirth/ neonatal ICU admission / poor APGAR/ Birth asphyxia? | Thank you very much for your clarification. This study excluded those having either undergone stillbirths or had their infants hospitalized due to serious delivery complication. Thus, the following sentence has been inserted accordingly: *“Those having either undergone stillbirths or had their infants hospitalized due to serious delivery complication (e.g. congenital diseases and cerebral palsy) were excluded from the samples for this study”.* (Line 189-191, page12). |
| line251  -253  Page21 | Did they ask the opinion or they just used the translated version for cognitive interview? Or the investigator interpreted from the interviewer? | Thanks for your clarification. Please find Line 200-208, page12 which describes the methods of cognitive interviewing. There are two types of cognitive interviewing approaches: (i) think aloud approach; and (ii) verbal probing approach. Of these two methods, verbal probing approach was employed for the items whose questions were confusing enough to require longer time to answer. To conduct cognitive interviewing, the cognitive interview guide was developed. At first, the pre-developed “proactive verbal probes” were used after each question such as: “What happened when [specific key question]?” (Recall probes); “How did you arrive at that answer?” (Process-oriented probe); “Why do you say…?” (Elaborative probes); “What does [a key term from the survey question] mean to you?” (Meaning-oriented probe); “Was this question difficult for you to answer?” (Evaluative probe); “How would you rephrase this question to make it better?”. Then, “reactive verbal probes” were used according to the respondent’s reactions. The verbal probing approach was useful to elicit respondent’s experiences behind the frequency response options; alternative keywords and their meanings; and the reason why they choose a given response option.  Here it means that we asked the initial Khmer translation reflected from expert’s suggestion, but it did not make sense among respondents. |
| line286  -287  Page23 | How did they ask about decision making? Did you ask them the indication for cesarian? how did you decide that they were involved in decision making or not? Were they given the liberty to refuse for cesarian? | Thanks for your clarification. We asked the respondents about their subjective experiences, by using the following question in Khmer. (back- translation of question in English) “Did you feel like the medical staffs at the facility considered your ideas in decisions about your care?”. Eleven of the 20 women who had vaginal deliveries were unfamiliar with the question intent because they have never thought or experienced their participation in selecting types of maternal health services. On the other hand, six respondents who had cesarean deliveries at the urban hospital stated the situation where they decided to have cesarean deliveries. Thus, we added the description of concrete and detailed process of cognitive interviewing (see Line 200-208). |
| line402  -403  Page30 | Was family involved in decision making? Or just woman and medical staff? | This comment is highly appreciated, because family’s involvement in decision making is important. However, the PCMC scale is the tool exclusively for asking women about their childbirth experiences. Thus, involvement of family members in the decision-making is beyond the scope of this study. |
| line405  -407  Page30 | Do you have any evidence in your interviews so that we can corelate the informed consent with karma? | Thanks for this comment. No valid narrative interview data could be obtained from the cognitive interviewing. This is a novel discussion part based on the author's experiential knowledge with 17 years of working experience in Cambodia using cited reference [49]. Note that our expert panel agreed with this discussion. |
| line415  -416  Page31 | Is it a “doubt” or health care providers are not aware and not recommending position of choice? | Thanks for this comment. In Cambodia, freestyle delivery has not been yet disseminated and practiced nationwide by healthcare providers. Thus, for both healthcare workers and pregnant women, supine position remains common. To make this point clearer, the sentence has been rephrased as *“This is because they have no doubt about giving birth in the supine position, as freestyle delivery has not been adequately known to women and practiced by medical staffs in Cambodia”*. (Line 431-433, page32) |
| line426  page32 | What kind of help did you ask for? | Thanks for your clarification. We asked subjective experiences of respondents without making a determination using following question in Khmer. (back- translation of question in English) “When you needed help, did you feel the medical staffs at the facility respond to what you need?”  To avoid confusion, the full text of Item #19*“when you needed help, did you feel the medical staffs at the facility respond to what you need?”* instead of “when you needed help from medical staff” has been amended to revised manuscript (Line 444-445, page33) |

[Response to Reviewer #2]

This is an honest study I believe. Authors have disclosed their limitations too. It is very much relevant for improving MCH service delivery at different levels of healthcare of a country. This study has thrown light up on some of the ignored areas of healthcare delivery system. This has got immense role in improving access and utilization of the public healthcare system by the citizens. This qualitative research is expected to help the national policy makers to rectify the loopholes if any.

- Thank you very much for your heart-warming comment. Although there were several limitations, we believe this study helps light up on some of the ignored areas of research and MCH service in Cambodia. I would like to keep working for women centered maternity care. Thank you.

[Response to Academic Editor]

Based on PLOS’ questionnaire on inclusivity in global research, subsections ‘Ethics’ (Line 226-233, page14) and ‘Inclusivity in global research’ (Line 235-238, page15) has been added to Methods section.

End
